# Xanthogranulomatous pyelonephritis with calculus migration into the psoas abscess: an unusual complication

**DOI:** 10.4322/acr.2020.200

**Published:** 2020-12-08

**Authors:** Manpreet Singh, Dillibabu Ethiraj, Venkatraman Indiran, Niranjan Dhanaji Kanase, Prabakaran Maduraimuthu

**Affiliations:** 1 Sree Balaji Medical College and Hospital, Department of Radio-diagnosis, Chennai, Tamilnadu, India

**Keywords:** Pyelonephritis, Xanthogranulomatous, Urinary Tract Infections, Escherichia coli

## Abstract

Xanthogranulomatous pyelonephritis (XGP) is a rare variant of chronic pyelonephritis. It is characterized by progressive parenchymal destruction caused by chronic renal obstruction due to calculus, stricture, or rarely tumor, resulting in kidney function loss. Herein, we describe the case of a 36-year-old female who presented with left loin pain, left lower limb pain, and dysuria. On contrast-enhanced computed tomography (CECT), multiple abscesses and an obstructive staghorn calculus were depicted in the left kidney with the classical appearance of “Bear Paw Sign.” An abscess with calculi was also present within the left psoas muscle. Though psoas muscle abscess in association with XGP was described, a ureteric fistula and calculi within the psoas muscle have not yet been reported in the literature. Left nephrostomy was performed, which came out to be positive for *E. coli* on culture. The patient underwent left nephrectomy, and the histopathological report of the surgical specimen confirmed XGP.

## INTRODUCTION

Xanthogranulomatous pyelonephritis (XGP) is a chronic granulomatous inflammatory disease of renal parenchyma, which is associated with urinary tract infection and obstruction. It is more frequently seen in middle-age females, and elderly.^1^ ^,^ ^2^ However, it has also been reported to occur in neonates. Patients usually present with abdominal pain, pyrexia, burning micturition, hematuria.^3^ In XGP, the normal renal parenchyma is infiltrated by xanthomatous histiocytes, lymphocytes, neutrophils, and multinucleated giant cells.^1^ ^,^ ^3^ Herein, we report a case of XGP associated with fistula of the ureter to the psoas muscle with multiple calculi. The patient went through a series of imaging modalities, surgical therapy, followed by histopathological correlation.

### Case Report

**Figure 1:**
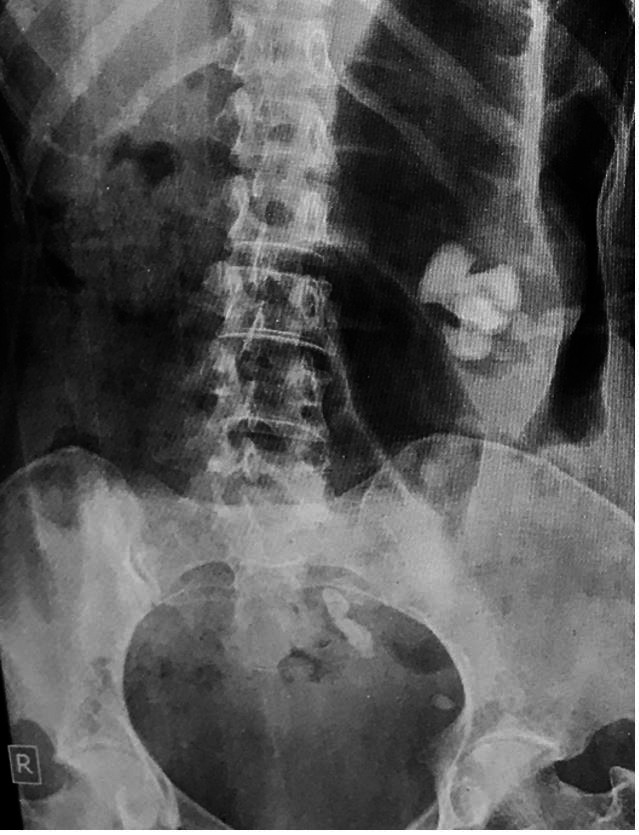
Abdominal plain radiograph shows a large radio-opaque shadow in the left renal fossa and multiple small radio-opaque shadows in the left pelvic region. These findings are consistent with multiple calculi.

A 36-year-old non-diabetic female presented to our hospital, complaining of left loin pain over the last 3 months, followed by dysuria and left lower limb pain. On examination, the patient was afebrile with pulse of 80 beats per minute, and blood pressure 130/80 mmHg. She exhibited a painful flexion of the left thigh and left loin tenderness. Laboratory parameters revealed leukocytosis WBC ~22.5 x 10^3^/µI (normal range: 5000-11000 /µL) and anemia - Hemoglobin of 8.2 g/dL (normal range: 11.5-16.5 g/dL). Renal Function test and serum electrolytes were within normal limits. The urinalysis revealed 10-15 leukocytes/HPF (normally < 5 leukocytes/HPF). On a plain radiograph, there was a large radio-opaque shadow in the left renal fossa (Figure 1).

**Figure 2:**
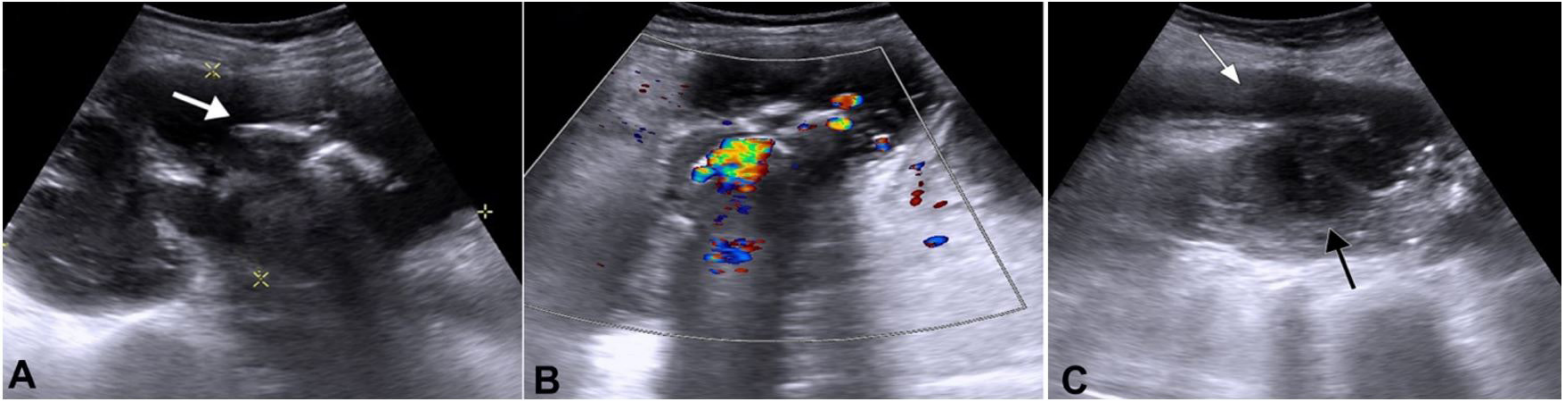
Abdominal USG showing a calculus and left psoas abscess. **A** and **B –** A large calculus probably a staghorn calculus (white arrow) with a twinkling artifact and posterior acoustic shadowing; **C –** Dilated proximal left ureter (white arrow) in close contact with a large collection in the left psoas region (black arrow).

The abdominal ultrasound (USG) showed a large calculus, probably a staghorn calculus measuring ~ 3.0 cm with a twinkling artifact and posterior acoustic shadowing (Figure 22B). Also, the proximal left ureter was dilated, measuring 1.76 cm and was seen draining into a large collection measuring 11.2 × 4.1 cm within the left psoas region (Figure 2C).

**Figure 3:**
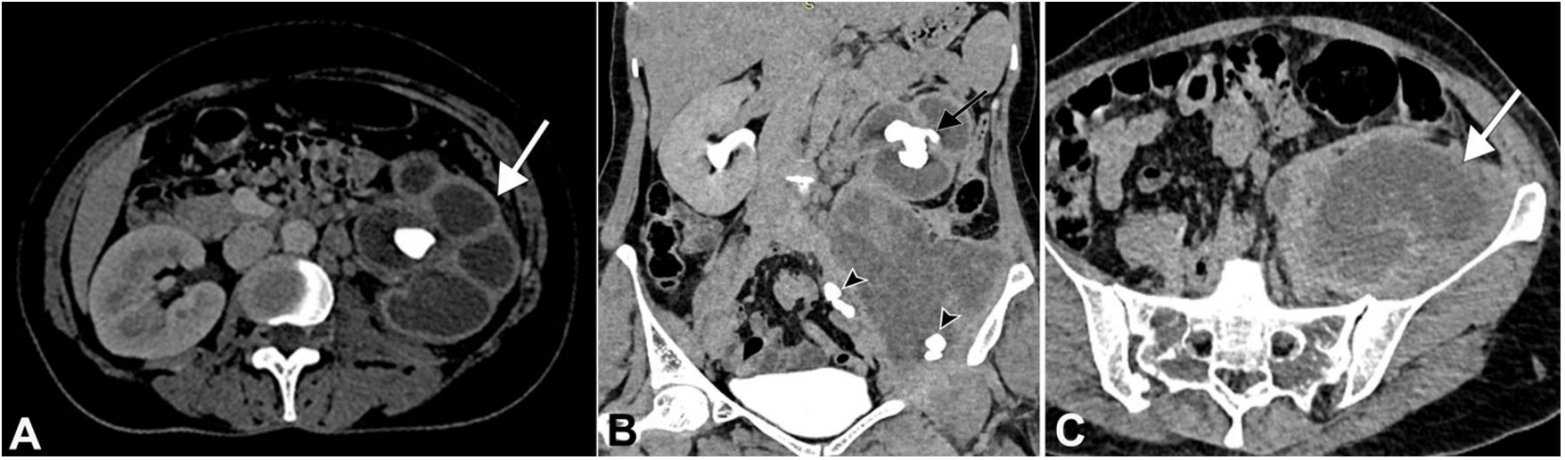
Abdominal CECT axial and coronal section shows XGP of the left kidney. **A** – Corticomedullary phase of CECT shows an enlarged left kidney with gross hydroureteronephrosis with thinning of renal cortex and a staghorn calculus within the renal pelvis, which is contracted, whereas calyces are dilated giving the appearance of a “Paw of A Bear” (white arrow); **B –** Excretory phase of CECT shows a staghorn calculus measuring 3.8 × 2.4 cm (black arrow) within the renal pelvis. Multiple calculi, the largest measuring 1.6 × 1.4 cm (arrowheads), were noted in the ureter and the collection; **C –** Large collection within the left iliopsoas muscle consistent with left psoas abscess (white arrow).

The CECT depicted an enlarged left kidney with gross hydroureteronephrosis with thinning of the renal cortex and a staghorn calculus measuring ~ 3.8 × 2.4 cm within the renal pelvis. The renal pelvis appeared contracted, whereas calyces were dilated, giving the appearance of a “Paw of a Bear,” referred to as “Bear Paw Sign” (Figure 3A). Also, a large collection measuring 13.0 × 4.0 cm was found along the left iliopsoas muscle harboring multiple images consistent with calculi, the largest measuring ~1.6 × 1.4 cm (Figure 33C).

The working diagnosis based on the imaging exams was left XGP with psoas abscess. Left nephrostomy was performed, and purulent fluid was drained, from which *Escherichia coli (E. coli)* was isolated on culture. After 5 days, a left-sided nephrectomy was performed, and the specimen was sent for histopathology examination. The surgical specimen weighed around 150 g (average 124g for female’s left kidney). The excised kidney measured 10.0 × 7.0 × 4.0 cm and was partially covered with perinephric fat. The attached ureter measured ~ 4.0 cm in length. The cut section of the kidney showed a staghorn calculus filling the pelvicalyceal system measuring 3.5 × 1.0 cm. Also, a few areas of yellowish discoloration were noted within the pelvicalyceal system, probably due to xanthoma cells (Figure 444C).

**Figure 4:**
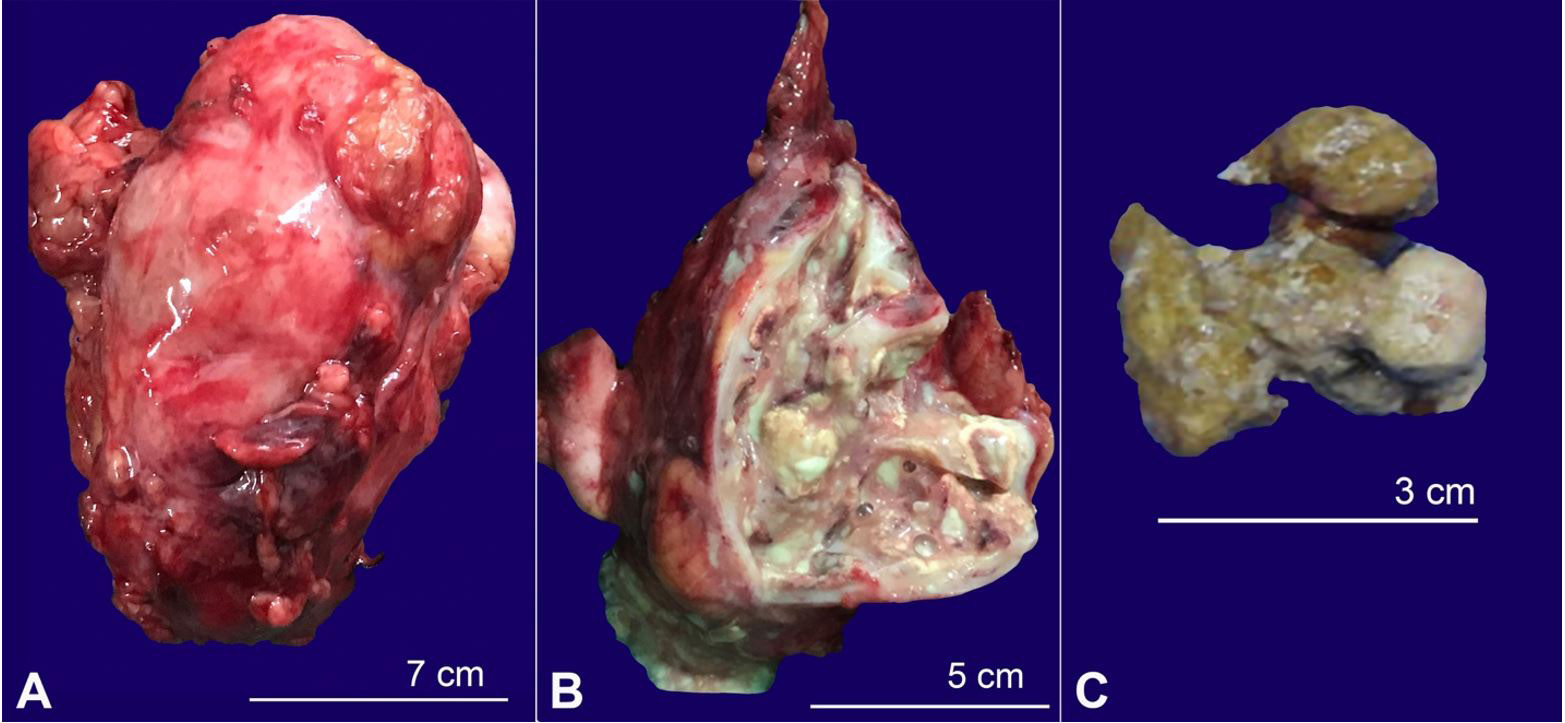
Gross findings of the excised left kidney. **A –** Gross view of the left kidney and attached ureter partially covered by perinephric fat; **B –** The cut surface of the left kidney. Note the presence of multiple calculi and necrotic cheesy material replacing the renal parenchyma with loss of cortico-medullary differentiation; **C –** The staghorn calculus.

**Figure 5:**
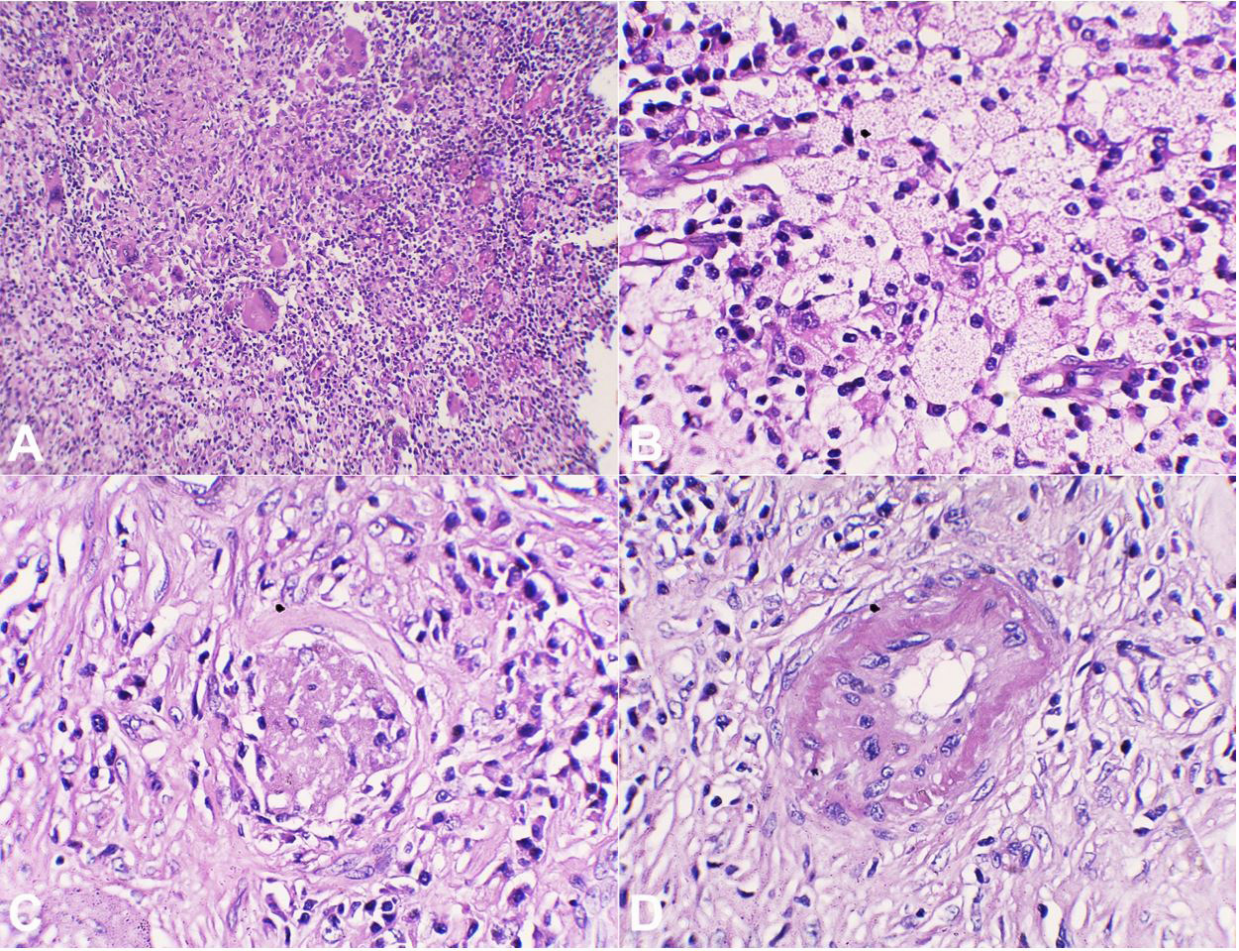
Photomicrographs of the left kidney. **A –** Microscopic section showing replacement of the renal parenchyma by sheets of acute and chronic inflammatory cells composed of neutrophils, lymphocytes, and plasma cells, scattered foreign body giant cells (H&E10X); **B and C –** Section shows sheets of polygonal histiocytes having abundant pale eosinophilic and foamy cytoplasm with a round nucleus and annexed with focal aggregates of lymphocytes (H&E 40X); **D –** Section shows the hyalinised glomeruli and thick-walled blood vessel (H&E 40X).

Microscopically, the kidney sections showed hyalinized glomeruli and atrophic tubules lined by flattened cells, and the surrounding blood vessels were thickly walled. Interstitial fibrosis with a dense collection of inflammatory cells composed of sheets of lymphocytes, plasma cells, neutrophils, histiocytes, and multinucleated giant cells were seen. Islands of foamy macrophages, foci of calcification, lymphoid follicles, and thickened capsule were also seen. At some places, the inflammatory cells and the proliferating capillaries were overlined by a transitional epithelium with ulceration. (Figure 5AD).

## DISCUSSION

XGP is a suppurative granulomatous infection characterized by progressive parenchymal destruction caused by chronic renal obstruction due to calculus or stricture or rarely tumor, resulting in a non-functioning kidney. XGP accounts for 0.6% to 1% of all pyelonephritis cases, globally. The common organisms on urine culture are *Proteus mirabilis, E coli, or Staphylococcus aureus.* The annual incidence of XGP is 1.4 per 100,000. XGP is a rare variant of pyelonephritis and is seen more commonly among females.^1^ ^,^ ^4^ The most common presenting symptoms are flank or abdominal pain, fever, pyuria, weight loss, hematuria. There are three types of XGP (i) diffuse, which is the most common type; (ii) segmental; and (iii) focal, which is limited to the cortex.^5^ ^-^ ^7^ Diffuse XGP is associated with massive renal enlargement, peri-pelvic fibrosis, hydronephrosis, lithiasis, and lobulated mass replacing the renal parenchyma. Preoperatively, it is difficult to differentiate, based on clinical and radiological features alone, XGP from other entities like tuberculosis and renal cell carcinoma (RCC). Hence, histopathological and immunohistochemistry correlation is required.^3^ The ultrasound of XGP typically demonstrates an enlarged kidney with dilated calyces and loss of cortico-medullary differentiation (CMD). In more than 70% of the XGP cases, renal calculus is present, which on USG appears as an amorphous echogenic structure and generally shows a posterior acoustic shadowing and twinkling artifact.^3^ In our case, the patient showed a large radiopaque shadow in the left renal fossa on the radiograph. The abdominal USG revealed large hyperechoic foci in the pelvicalyceal system of the left kidney with posterior acoustic shadowing and twinkling artifact, which is suggestive of calculus.

CT is the gold standard diagnostic method for XGP because it demonstrates the specific intrarenal findings and shows the extrarenal extension, which is useful for surgical scheduling.^8^ ^,^ ^9^ In our case, the CECT showed an enlarged kidney with low-density areas within the renal parenchyma and a large obstructive staghorn calculus causing hydroureteronephrosis. The low-density areas were suggestive of an abscess or fluid. “Bear paw sign” is better appreciated on the corticomedullary phase of CECT.^4^ ^,^ ^5^ Also, a collection containing multiple calculi was noted within the left iliopsoas muscle. This is an unusual finding in XGP presenting with psoas abscess and, according to our knowledge, has not yet been reported in the literature. Due to the presence of ureteric calculus, chronic obstruction with concomitant infection is likely to have caused significant damage to the ureteric wall and resultant fistula, and urinoma formation within the psoas muscle. Subsequent secondary infection of the urinoma could have resulted in the psoas abscess formation. The drained abscess was positive for *E coli,* which is the most frequent bacterial cause of urinary tract infection.^10^

The patient was prescribed broad-spectrum antibiotics for 5 days, followed by total left nephrectomy. Usually, on MRI, XGP appears as a hyper signal on T1- weighted image due to the lipid-laden foamy macrophages and shows a signal void if calculi are present within the collecting system. However, this MRI sign is not always reliable if the lesion lacks a certain amount of xanthoma cells.

Medical and surgical management plays an important role in the treatment of XGP. In cases of unilateral diffuse XGP, nephrectomy is the usual therapeutic option. While in cases of bilateral XGP, when surgery is not feasible or in patients with focal XGP, the broad-spectrum antibiotic regimen is indicated.^3^ However, Leoni et al. observed a mortality rate of 10% in a series of 10 patients, despite the nephrectomy.^11^ Since our case could not be managed conservatively with only on antibiotics regimen, the abscess drainage and a left-sided nephrectomy were necessary to prevent the development of a septic shock. In cases of absence of the typical signs like staghorn calculus, the differential diagnosis of XGP, based strictly on imaging, includes renal tuberculosis, renal cell carcinoma, renal abscess, and renal angiomyolipoma. Hence, in these cases, the histopathological examination is necessary. A partial or total nephrectomy should be performed with the histopathological diagnostic confirmation, in the cases where the biopsy is not feasible.^3^ ^,^ ^12^ XGP, generally presents with replacement of the cortico-medullary junction by soft yellow nodules, along with filling of the calyces with pus and debris. On the histological examination, there is diffuse infiltration of the renal parenchyma with plasma cells, histiocytes, lymphocytes, neutrophils, multinucleated giant cells, and lipid-laden macrophages (xanthoma cells). The xanthomatous cell of XGP sometimes resembles the clear cells of RCC. Unlike that of xanthoma cells, the cytoplasm of tumoral cells is clearer, which has a foamier cytoplasm. Therefore, Immunohistochemistry (IHC) study may be helpful in certain cases.^3^ ^,^ ^6^

## CONCLUSION

XGP is an uncommon entity associated with urinary tract obstruction and infection. Also, its association with calculus migration into psoas abscess is an unusual complication. It is difficult to differentiate it from renal cell carcinoma on imaging, and hence histopathological correlation is required. Imaging features are not enough to confirm the diagnosis of XGP, but in this case, demonstration of “Bear paw sign” helped in ruling out other uncommon differentials. In this case, the correlation of the clinical findings with radiologic features, followed by proper treatment and histopathological correlation not only helped in confirming the disease but also improved patient recovery.
